# The Late Positive Potential as a Reliable Neural Marker of Cognitive Reappraisal in Children and Youth: A Brief Review of the Research Literature

**DOI:** 10.3389/fpsyg.2020.608522

**Published:** 2021-02-17

**Authors:** Heather Kennedy, Tina C. Montreuil

**Affiliations:** ^1^Department of Educational and Counselling Psychology, Faculty of Education, McGill University, Montreal, QC, Canada; ^2^Department of Psychiatry, Faculty of Medicine, McGill University, Montreal, QC, Canada; ^3^Research Institute, Montreal University Health Centre, Montreal, QC, Canada

**Keywords:** emotion regulation, cognitive reappraisal, electroencephalogram, late positive potential, child and youth mental health

## Abstract

The mental health of young people is a growing public health concern. With socio-emotional difficulties in youth often resulting in psychiatric disorders later in life and most with mental health conditions rather stabilizing in time, it is essential to support healthy socio-emotional development. With a comprehensive definition of mental health, since emotion regulation (ER) plays a critical role in prevention, it becomes imperative to better understand how children effectively manage their emotions from an early age. Determining effective use of ER skills relies on adequate measurements. Typical methods of data collection in children present consistent shortcomings. This review addresses research findings considering the suitability of the late positive potential measured through electroencephalogram as a neural indicator of ER in children and youth. There is growing evidence, as reported in this review, that indicates that the late positive potential may be a reliable neural indicator of children's cognitive reappraisal abilities more specifically. Results generally suggest that the late positive potential amplitudes are sensitive to directed reappraisal in children. However, given the scant research, questions remain regarding developmental trends, methodology, interindividual variability, reappraisal of various stimuli, and how the late positive potential may relate to more traditional measures of ER. Directions for future research are provided, which are expected to address unanswered research questions and fill literature gaps. Taken together, the findings reviewed indicate that the late positive potential is generally sensitive to directed cognitive reappraisal in children and that there is promise of establishing this neural marker as an indicator of ER.

## Background

The mental health of young people is a growing public health issue (Patel et al., 2007). It is essential for youth to have the best mental health in order for them to achieve optimal functioning (Chadda, 2018). Approximately 10–20% of children and adolescents are affected by mental health problems worldwide (Kieling et al., 2011). In Canada, like the rest of North America, current estimates suggest that ~14% of children between the ages of 4 and 17 (over 800,000 children) have clinically relevant mental health problems that cause significant anguish and impairment across multiple life domains (Waddell et al., 2005, 2007). Even more alarming is the fact that only 20% of young children with mental health problems receive treatment, leaving a substantial 80% without mental health care services (Martini et al., 2012). Social and emotional difficulties in childhood increase the risk of developing psychiatric disorders, depression, anxiety, and substance use disorders in later life (Costello et al., 2004; Stansfeld et al., 2008). It is therefore essential to promote positive social and emotional learning (OECD, 2015) and prevent mental health problems among children and adolescents (Viner et al., 2012), especially given the stability of most psychiatric disorders (GBD, 2017) in the absence of targeted preventive actions and intervention. A better understanding of the variables that should become the focus of prevention efforts is key for the development of emotional resilience, a known protection factor against mental health disorders (Compas et al., 2017; Gross et al., 2019; Daniel et al., 2020).

## Emotion Regulation and Mental Health

Emotion regulation (ER) is an important component of mental health (Gross and Muñoz, 1995). Children as young as 8 or 9 years use cognitions or thoughts about themselves, their feelings, or others to manage their emotions (Harris et al., 1989; Terwogt and Stegge, 1995; Saarni, 1999). In turn, the ability to successfully and adaptively regulate our emotions is known to have positive outcomes in many domains of life, including physical health, well-being, and academics (John and Gross, 2004). Taken together, the regulation of both positive and negative emotions can hence be protective of an individual's overall mental health well-being. Conversely, research supports the association between greater engagement in maladaptive ER strategy use and psychopathology [see Schäfer et al. (2017), for a meta-analysis]. Cognitive ER, defined as the conscious thoughts one uses to manage an emotional response to an aversive event (Thompson, 1991; Garnefski et al., 2001; Gross, 2001), is of particular interest when examining ER and mental health (Hu et al., 2014) along a comprehensive approach. Moreover, gaining a greater understanding of cognitive ER strategies has important implications for preventive and intervention efforts. As such, identifying a robust and valid methodology to measure improvements in ER is critical in determining treatment outcomes (McLean et al., 2020).

## Emotion Regulation

ER refers to the use of strategies that seek to regulate the occurrence, intensity, and expression of one's emotions (Thompson, 1994; Gross, 1998). According to the process model of ER, emotions can be controlled and modulated at various stages of the emotion generative process through the use of various strategies (Gross, 1998). ER strategies can be antecedent or response focused such that we have the ability to utilize strategies before the emotion response has been fully activated (antecedent focused) and after the emotion is already underway (response focused) (Gross, 2002). Further, these strategies are generally conceptualized as being either adaptive or maladaptive. Adaptive ER strategies are consistently linked to beneficial long-term outcomes and include strategies such as cognitive reappraisal or problem solving. Maladaptive strategies, on the other hand, are consistently associated with negative long-term outcomes and include strategies such as avoidance and rumination (Aldao et al., 2010). The ability to regulate one's emotions thus has clear implications for coping and reacting to adversity, which in turns has implications on mental health and well-being (Montreuil and Tilley, 2017).

## Current Trends in Data Collection

Observational methods have commonly been used when examining the ER of youth given consistent concern that younger children have difficulty in reflecting on and reporting their emotions. Observational studies seek to address this concern by using trained raters to assess the child's ER in various tasks typically completed in a laboratory setting in which a negative emotion is induced (i.e., frustration). However, this then introduces concerns for generalizability since researchers rely on making these observations under controlled conditions that generally lack ecological validity (Cole et al., 2004).

To address this concern, researchers more typically rely on the use of questionnaires which are both cost-effective and easy to administer. Only the individual is truly able to assess and integrate a variety of information about their own emotional experiences (Adrian et al., 2011). However, again, concerns about the child's ability to reflect on and quantify their emotional experiences also apply to this methodology (Zeman et al., 2007). Many researchers also utilize multiple informants to assess children's ER such as parents and teachers given their increased emotional intelligence. Still, concerns regarding biases and other processes have been noted as having the potential to affect these raters' assessment (Fergusson et al., 1993), since multi-informants can also respond differently (Rabinowitz et al., 2018). Given these limitations, more objective approaches to data collection are becoming ever more common in ER research conducted with children. Event-related brain potentials (ERPs) recorded through electroencephalography (EEG) are best to estimate the neural processes elicited ensuing emotional processing (McLean et al., 2020).

## Understanding Emotion Regulation Using EEG

An EEG is a test that measures brain activation through noninvasive procedures by picking up electrical signals produced by the brain's neurons. Brain activity produces electrical signals that are recorded using electrodes. These signals are detected and averaged over numerous trials of the same activity to create ERPs. Differential patterns in these electrical signals during both resting and induced emotional states have been successfully used to predict ER abilities in children, as well as to predict anxiety and other related mood disorders (e.g., McManis et al., 2002; Hannesdóttir et al., 2010). Further, there are certain brain activation responses that have been associated with emotional responding and ER [e.g., late positive potential (LPP)]. Thus, these patterns of brain activity are frequently investigated as predictors of mood disorders and thereby will serve as indicators of ER abilities (Thibodeau et al., 2006).

## Neural Signature of Emotion regulation: Late Positive Potential

The LPP is an ERP which can be described as a slow, positive-going waveform that emerges around 200–300 ms after stimulus onset (Hajcak and Dennis, 2009; Hajcak et al., 2010; Kujawa et al., 2012). LPP reflects the processing of and attention facilitated to emotional stimuli such as distressing visual images versus more neutral stimuli (Schupp et al., 2004; Herbert et al., 2008). LPP has been suggested to be a useful tool in examining cognitive reappraisal (Hajcak and Niwuwenhuis, 2006; Foti and Hajcak, 2008; Hajcak et al., 2010; MacNamara et al., 2011). Reappraisal involves changing the emotion meaning and significance of an emotion-inducing stimulus (Gross and John, 2003; Ochsner and Gross, 2005; Foti and Hajcak, 2008). For example, before a person visits their sick pet at the veterinarian's office, they may focus on the idea that their pet is there to recover and is receiving necessary care in order to be healthy again. Through this reappraisal process, the negative emotions which would typically be associated with a negative appraisal of “going to see a sick pet” is lessened before the emotional stimulus is internalized as being negative. In adult samples, a number of researchers have shown that LPP amplitudes are reduced when participants are instructed to reappraise unpleasant stimuli (Foti and Hajcak, 2008; MacNamara et al., 2011). As such, relying on LPP for measuring ER in youth is promising as it serves as a more objective approach given that it reduces the influences of bias. However, there still remains many unanswered questions surrounding the generalizability of these results to youth populations.

## Current Research Base: Reappraisal and LPP in Children

ERPs have been posited to be reliable and meaningful indices of ER in children (Dennis, 2010), and it has been more recently suggested that neural markers of childhood psychopathology could be used to help improve the therapeutic outcome by informing the improvement of diagnostic tools and development and validation of novel interventions (McLean et al., 2020). Taken together, the present review explored research findings relating to the evaluation of LPP as a suitable neural indicator of cognitive reappraisal in youth. A summary of the current research base is presented in Table 1. This brief review of literature yielded 10 articles from three unique countries and one review. The earliest published article on LPP and reappraisal in children was published in 2009, and the most recent was published in 2019. The majority of included articles were published after 2014. Included studies relied on similar study designs stemming from early works by Dennis and Hajcak (2009) and DeCicco et al. (2012). This included articles sampling a broad age range of children from preschool age through to age 15. The largest number of studies included children from early adolescence (age 8–12).

**Table 1:** Summary of the research base: reappraisal as measured by LPP in children and youth (in chronological order).

**Author(s) (year)**	**Country**	**Sample**	**Reappraisal procedure**	**Summary of results**	**Effect sizes relevant to reappraisal LPP**
Dennis and Hajcak (2009)	USA	20 5–10-year-olds (boys *M_*age*_* = 85.60 months, *SD* = 14.95; girls *M_*age*_* = 90.00 months, *SD* = 21.24), 50% female	Unpleasant images presented for 2,000 ms followed by audio of negative or neutral interpretation (5,000–7,000 ms). The same picture was presented again for 2,000 ms. Each of the pictures was presented twice along with the same interpretation.	LPP amplitudes to unpleasant pictures were decreased when pictures were reappraised in a neutral instead of negative way. This effect was significant in the middle window for all participants with the exception of girls aged 5–6.	Effect of interpretation type × gender × age (middle window) $\eta_{{\text{p}}^{2}}$ = 0.24 Effect of interpretation type × gender × all spatial dimensions (middle window) $\eta_{{\text{p}}^{2}}$ = 0.36
DeCicco et al. (2012)	USA	32 5–7-year-olds (*M_*age*_* = 76.56 months, *SD* = 6.17), 34% female	Audio negative or reappraisal story followed by a 500 ms delay. Unpleasant images then presented for 2,000 ms. Each of the pictures was presented twice along with the same interpretation. Participants were then presented with a neutral block with neutral stories and neutral images.	Negative stories and reappraisals generated larger LPP amplitudes when compared to the neutral baseline condition; however, LPPs did not differ between negative and reappraisal story conditions. Emotional content rather than reappraisal modulated LPP amplitudes.	Effect of condition (posterior region) η^2^ = 0.28 Effect of condition (central region) η^2^ = 0.22 Effect of condition (anterior region) η^2^ = 0.17
DeCicco et al. (2014)	USA	26 7–9-year-olds (*M_*age*_* = 98.42 months, *SD* = 6.04), 42% female (DeCicco et al., 2012 participants, 2 years later)	First passively viewed unpleasant, pleasant, and neutral stimuli for 2,000 ms each with a 1,500 ms interval. For the reappraisal task, negative or reappraisal stories were presented prior to stimulus onset with 500 ms delay. Then unpleasant stimuli were presented (2,000 ms). The other block consisted of neutral images and stories. Blocks were repeated twice.	LPP amplitudes were not reduced when comparing the reappraisal condition with the negative story condition. Positive correlations between increasing age and LPP sensitivity suggest that reappraisal may increase linearly between the ages of 7 and 9 years. LPPs for the unpleasant passive viewing condition were larger than LPPs for the unpleasant reappraisal stimuli, suggesting that asking children to reappraise had a regulatory effect.	Effect of condition (early window) η^2^ = 0.26 Effect of condition (middle window) η^2^ = 0.41 Effect of condition (late window) η^2^ = 0.46
Babkirk et al. (2015)	USA	32 5–7-year-olds (*M_*age*_* = 76.0 months, *SD* = 6.48), 34% female [from DeCicco et al. (2012) study]	Unpleasant images (2,000 ms) preceded by a negative or reappraisal story with a 1,500 ms interval. Neutral pictures were also presented and paired with neutral stories. After each story, there was a 500 ms delay prior to stimulus onset. Blocks were repeated twice.	LPP amplitudes were not significantly reduced in the reappraisal vs. negative conditions. Due to great variability at T1, children were divided into positive (LPP smaller for reappraisal vs. negative) and negative (LPP greater for reappraisal vs. negative) LPP difference groups. The positive reappraisal group showed smaller LPP amplitudes following reappraisal versus the negative story condition.	N/A
Hua et al. (2015)	China	20 48–71-month-olds (*M_*age*_* = 59.60 months, *SD* = 8.06), 40% female	Unpleasant pictures (3,000 ms) preceded by negative or neutral audio interpretations (2,000 ms, 4 Chinese characters, 800–1,000 ms delay). Following the instruction for interpretation, participants repeated these and reported if they understood the meanings.	LPP amplitudes following neutral interpretations were significantly reduced compared to those which followed negative interpretations.	Effect of condition (posterior region) $\eta_{{\text{p}}^{2}}$ = 0.75 Effect of condition (central region) $\eta_{{\text{p}}^{2}}$ = 0.69 Effect of condition (anterior region) $\eta_{{\text{p}}^{2}}$ = 0.45
Leventon and Bauer (2016)	USA	28 8-year-olds (*M_*age*_* = 8.48 years, range = 8.09–8.84), 100% female	Positive, neutral, and negative images (2,000 ms) followed by audio narratives (4,500–7,000 ms) that matched the image or reappraised in a neutral way. The picture was then presented for a second time.	Immediately after manipulation and after a delay, LPPs to reappraised negative stimuli were reduced.	Immediate effect of condition (central cluster, second window) $\eta_{{\text{p}}^{2}}$ = 0.17 Delayed effect of condition (all windows in each cluster) $\eta_{{\text{p}}^{2}}$s > 0.28
Van Cauwenberge et al. (2017a)	Belgium	ADHD: 18 8–12-year-olds (*M_*age*_* = 9.8 years, *SD* = 1.5), 22% female TD: 24, 8–12-year-olds (*M_*age*_* = 9.8 years, *SD* = 1.4), 25% female	Unpleasant images (2,000 ms) followed by an auditory neutral or negative interpretation (5,000–10,000 ms). The same picture was presented again. Each of the pictures was presented twice along with the same interpretation.	Groups differed for LPP modulation, suggesting that the ADHD group showed less positive modulation in the LPP during reappraisal. However, the main effect of condition on LPP was not significant, either over groups or within groups separately.	Effect of group η^2^ = 0.12 Effect of group × condition η^2^ = 0.12
Van Cauwenberge et al. (2017b)	Belgium	60 8–11- and 12–15-year-old groups (8–11 boys *M_*age*_* = 9.40 years, *SD* = 1.06; 8–11 girls *M_*age*_* = 9.40 years, *SD* = 1.06; 12–15 boys *M_*age*_* = 13.67 years, *SD* = 1.18; 12–15 girls *M_*age*_* = 13.53 years, *SD* = 1.19)	Unpleasant images (2,000 ms) followed by an auditory negative or neutral interpretation (5,000–10,000 ms). The same picture was presented again. Each of the pictures was presented twice along with the same interpretation.	LPP amplitudes were reduced after reappraisal of negative images in the 12–15-year-old group, but similar effects were not found for 8–11-year-olds (further attributed to the youngest children). There was a significant linear increase of LPP modulation with age.	Effect of condition × age group (early window) η^2^ = 0.08 Effect of condition in older children (early window) η^2^ = 0.24
Liu et al. (2019)	China	46 8–12-year-olds (*M_*age*_* = 119.72 months, *SD* = 15.57), 48% female	Neutral and pleasant images (1,500 ms) preceded by audio neutral interpretation for neutral images and audio neutral or positive interpretations of the pleasant images (4,000 ms). Each picture was displayed twice.	Children were able to upregulate positive emotions through reappraisal. The LPP amplitudes following upregulation were larger than those following the neutral interpretations. Further, pleasant pictures elicited larger LPP amplitudes compared to neutral pictures.	Effect of condition (early window) $\eta_{{\text{p}}^{2}}$ = 0.18. Effect of condition (middle window) $\eta_{{\text{p}}^{2}}$ = 0.32. Effect of condition (late window) $\eta_{{\text{p}}^{2}}$ = 0.19
Myruski et al. (2019)	USA	116 5–7-year-olds (*M_*age*_* = 6.01 years, *SD* = 0.74), 46% female	Unpleasant and neutral images (2,000 ms) preceded by a negative or reappraisal story for unpleasant images and a neutral story for neutral images. Children had their parent either absent (audio-recorded story presented twice), present (audio-recorded story presented twice), or scaffolding (parent-guided reappraisal).	LPP amplitudes significantly reduced in the reappraisal condition compared to the negative condition. Reappraisal-induced reduction in LPP was enhanced in US and Japanese cultures when parents scaffolded child ER. A reappraisal effect was also found when parents were merely present during the task.	Effect of social context group $\eta_{{\text{p}}^{2}}$ = 0.11 Effect of culture $\eta_{{\text{p}}^{2}}$ = 0.04
**Review**	**Summary**		**Future directions**		
Dennis (2010)	There is preliminary evidence that modulation of the LPP by way of cognitive ER may be a clinically relevant marker of ER in children and may affect dysregulation and behavior.		Explore diverse paradigms, particularly those in which emotional processing directs cognition. Assess changes in emotion in relation to task performance (highly emotional vs. little emotion). Development of ER assessment task battery to increase generalizability. ERP markers of ER for more complex emotional behaviors. Examination of individual differences relevant to development of mood and behavior problems.		

### Across Development

Dennis and Hajcak (2009) suggested that the sensitivity of LPP to cognitive reappraisal undergoes a developmental shift. An upward trend in sensitivity of LPP modulation with age was also observed in the majority of articles, suggesting that the ability to use reappraisal may increase between ages 8 and 12 (DeCicco et al., 2012, 2014; Leventon and Bauer, 2016; Van Cauwenberge et al., 2017a,b; Liu et al., 2019). This shift has been consistently tied to the dependency of ER development on the maturation of executive functions with age as well as the development of cognitive changes such as those theorized by Piaget (1976). These processes may thus enhance the child's ability to exercise cognitive control, which is required for effective reappraisal (DeCicco et al., 2014).

Conversely, Hua et al. (2015) suggested that the common methodology shared among these studies may be the cause of lack of effects of reappraisal on LPP seen in younger children. Specifically, Hua et al. (2015) suggested that the 5–7-s-long guided interpretations used by many researchers may be too complex for young children. The children in these studies had to store long interpretations in their working memory, and as such, this may have impacted their ability to also reappraise the image that followed. To address this concern, Hua et al. (2015) simplified interpretations to be <2 s and four Chinese characters long. Through this approach, significant reductions in LPP modulations following reappraisal were found in preschoolers (4–5 years). Liu et al. (2019) also utilized shorter audio interpretations (4 s) and found similar reductions in LPPs in school-aged children (8–12 years).

Consequently, lack of effects in young children cannot be exclusively tied to methodological limitations. Even without any methodological adjustments, Myruski et al. (2019) found reappraisal-induced reductions in LPPs in children as young as 5 years old. Babkirk et al. (2015) separated their 5–7-year-old participants based on their LPP patterns (detailed below), finding that LPPs were smaller for reappraised stimuli compared to negatively appraised stimuli. Indeed, in other areas of ER research, children as young as 3 years old have been observed to be able to use a variety of strategies including cognitive reappraisal when frustrated (Stansbury and Sigman, 2000). Taken together, it is likely that preschoolers are able to use reappraisal to some degree to manipulate negative emotions. Yet, from the current research available, there remains much uncertainty regarding the developmental trends as evident in LPP.

### Interindividual Variability

Interindividual variability in LPP amplitudes is a rising concern that was noted in two of the research studies scoped, such that some participants experienced smaller LPPs while others experienced larger LPPs during reappraisal when compared to the LPPs from negative interpretations (Babkirk et al., 2015; Van Cauwenberge et al., 2017a). This interindividual variability could be contributing to the lack of consistent main effects of reappraisal in LPP. Van Cauwenberge et al. (2017a) proposed that this variability may be related to a number of factors, one of which being age. Babkirk et al. (2015) addressed this variability directly by separating their participants based on their LPPs for reappraisal in relation to those for negative interpretations. Findings indicated that those who had smaller LPPs for reappraisal stories over negative stories showed smaller LPP amplitudes following reappraisal. These individual differences were suggested to be related to the role of social context in developmental trajectories of ER. It was hence suggested that some participants were able to reappraise in an unfamiliar lab setting, whereas others may have required a more ecologically relevant context to rely on reappraisal (Babkirk et al., 2015).

### Regulation of Positive Stimuli

Experiencing positive emotions promotes one's well-being and mental health (Hu et al., 2014). As such, the upregulation of positive emotions is important to consider when examining ER holistically. To date, a single study has investigated reappraisal in children using LPP as a marker and found that LPP amplitudes were larger when 8–12-year-olds were guided to upregulate a pleasant image (Liu et al., 2019). However, few conclusions can be drawn from this one study, due to the consistent focus on the downregulation of negative stimuli within this field.

### Relation to Traditional ER Measures

Only four studies examined the relation between LPP and traditional measures of ER in youth. Van Cauwenberge et al. (2017b) found that LPP modulation did not correlate with self-reported reappraisal. Van Cauwenberge et al. (2017a) found a positive correlation between the LPP and a self-reported measure of cognitive reappraisal; however, this was determined to be driven by outliers. Still, the results of Dennis and Hajcak (2009) and Babkirk et al. (2015) support a relationship between informant (maternal) reports, observations of ER, and LPP modulation. As such, there remains much uncertainty regarding how LPPs may translate to questionnaires or observed ER, compromising the validity of both LPPs and traditional ER measures as markers of reappraisal. As has been well validated in adult population studies, more studies are required to investigate how LPPs can be manipulated to measure ER processes in childhood (Hajcak et al., 2010).

## Directions for Future Research

Most fundamentally, robust research in the area of ER in youth requires the implementation of rigorous methodology toward increased ecological validity of lab-directed reappraisal. In the scholarly articles screened for this brief review, reappraisal was provided by the experimenter (or through the parent) in all studies, making it difficult to infer that in fact children's own reappraisal capabilities were reflected through the reported LPPs. Rather, a more traditional methodology appears to explore how susceptible children are to the effects of induced or directed reappraisal. Further, several studies followed a paradigm not conducive to the definition of cognitive reappraisal as an antecedent-focused strategy as theorized by Gross (1998). Gross' (1998) definition of reappraisal specifies that this process occurs prior to the experience of emotion. Dennis and Hajcak (2009), Leventon and Bauer (2016), Van Cauwenberge et al. (2017a), and Van Cauwenberge et al. (2017b) all first presented their visual stimuli, followed by the audio interpretation, and then the stimuli were presented again. Given that these studies employed reappraisal after the emotional response was experienced, the results of these studies should be interpreted with caution given that they may have captured a response-focused regulatory attempt as opposed to the anticipated “reappraisal.” Future studies are encouraged to explore evaluation of children's own reappraisal capabilities by first teaching/coaching the child on how to reappraise independently before viewing unpleasant stimuli and then subsequently exploring how these amplitudes contrast with their self-reported reappraisal.

More recently, Myruski et al. (2019) had explored reappraisal using an increased ecological approach by having the child's parent present during the reappraisal task where they were sitting behind the child or guiding them through reappraisal by modeling. Children achieve successful ER partially through socialized regulation of emotion by caregivers, so methodology that neglects this influence may not be as ecologically relevant (Cabecinha-Alati et al., 2019, 2020; Myruski et al., 2019). Future studies are encouraged to explore how other social influences may impact reappraisal as evident in the LPP, such as the influence of a caregiver that provides encouraging or discouraging statements while the child engages in the reappraisal task.

Further, only one study from the extracted research base had investigated these emotional processes using special populations (ADHD; Van Cauwenberge et al., 2017a). LPP was found to be related to maternal reports of child anxiety (DeCicco et al., 2012) and broad symptoms of anxiety–depression (Dennis and Hajcak, 2009; DeCicco et al., 2014). Given the connection between ER and mental health, it is important to more directly examine LPP of youth with mental health challenges such as anxiety or depression. Through this, researchers may be able to detect patterns of LPP that may be indicative of mental health challenges or risks in youth. Considering this comprehensive approach to defining mental health, future studies should seek to gather LPPs of clinically anxious or depressed children and their counterparts in an effort to pinpoint these patterns.

Finally, given the scant research literature and inconsistency among results, replication and advancements in methodology are necessary to gather a more reliable understanding of LPP and reappraisal across ages, special populations, ethnicities, differing stimuli, etc. Future studies are especially encouraged to include younger samples given the widespread inconsistencies regarding LPP in this age group. Further, to attain a more holistic understanding of ER on a biophysiological level, researchers should continue to explore additional neural indicators, which may be indicative of reappraisal or other cognitive ER strategies. Through this, informed conclusions can be drawn regarding the biophysiological nature of ER across childhood, which in turn inform our broader understanding of childhood socio-emotional development.

## Conclusion

In conclusion, the findings disseminated within this brief review generally support the notion that LPP is sensitive to directed cognitive reappraisal in children. However, there remains many unanswered questions regarding the suitability of the LPP as a neural marker of cognitive reappraisal in children, which can be tied to this small research base and lack of replicable studies, highlighting the need for longitudinal studies examining both EEG and observational data across multiple timepoints in childhood to determine directionality. Nonetheless, the studies included in this review provide a strong foundation from which future studies can build upon. These results highlight the necessity of continued development and replication of studies, in order to make reliable conclusions about the soundness of LPP in measuring reappraisal throughout various stages of development. Nevertheless, EEG has great potential to increase the scientific rigor of ER research and, in turn, is expected to be clinically useful in the detection of ER difficulties as well as validation of intervention program effectiveness. In turn, such findings would have great implications on the promotion of mental health and well-being of children and youth.

## Author Contributions

All authors listed have made a substantial, direct and intellectual contribution to the work, and approved it for publication.

## Conflict of Interest

The authors declare that the research was conducted in the absence of any commercial or financial relationships that could be construed as a potential conflict of interest.
